# 24-hour central aortic systolic pressure and 24-hour central pulse pressure are related to diabetic complications in type 1 diabetes – a cross-sectional study

**DOI:** 10.1186/1475-2840-12-122

**Published:** 2013-08-27

**Authors:** Simone Theilade, Maria Lajer, Tine Willum Hansen, Christel Joergensen, Frederik Persson, Gudbjörg Andrésdottir, Henrik Reinhard, Stine Elkjær Nielsen, Peter Lacy, Bryan Williams, Peter Rossing

**Affiliations:** 1 Steno Diabetes Center, Niels Steensensvej 1, 2820 Gentofte, Denmark; 2 The Institute of Cardiovascular Science and National Institute for Health Research Biomedical Research Centre, University College London, London, UK; 3 Aarhus University, Aarhus, Denmark; 4 University of Copenhagen, Copenhagen, Denmark

**Keywords:** Ambulatory blood pressure, Brachial systolic blood pressure, Central aortic systolic pressure, Central pulse pressure, Diabetic complications, Type 1 diabetes

## Abstract

**Background:**

Non-invasive measurements of 24 hour ambulatory central aortic systolic pressure (24 h-CASP) and central pulse pressure (24 h-CPP) are now feasible. We evaluate the relationship between 24 h central blood pressure and diabetes-related complications in patients with type 1 diabetes.

**Methods:**

The study was cross-sectional, including 715 subjects: 86 controls (C), 69 patients with short diabetes duration (< 10 years), normoalbuminuria (< 30 mg/24 h) without receiving antihypertensive treatment (SN), 211 with longstanding diabetes (≥ 10 years) and normoalbuminuria (LN), 163 with microalbuminuria (30-299 mg/24 h) (Mi) and 186 with macroalbuminuria (> 300 mg/24 h) (Ma).

24 h-CASP and 24 h-CPP was measured using a tonometric wrist-watch-like device (BPro, HealthStats, Singapore) and derived using N-point moving average.

**Results:**

In C, SN, LN, Mi and Ma mean ± SD 24 h-CASP was: 114 ± 17, 115 ± 13, 121 ± 13, 119 ± 16 and 121 ± 13 mmHg (*p* < 0.001); and 24 h-CPP: 38 ± 8, 38 ± 7, 44 ± 10, 46 ± 11 and 46 ± 11 mmHg, (*p* < 0.001).

Following rigorous adjustment (24 h mean arterial pressure and conventional risk factors), 24 h-CASP and 24 h-CPP increased with diabetes, albuminuria degree, previous cardiovascular disease (CVD), retinopathy and autonomic dysfunction (*p* ≤ 0.031).

Odds ratios per 1 standard deviation increase in 24 h-CASP, 24 h-CPP and 24 h systolic blood pressure (24 h-SBP) were for CVD: 3.19 (1.68-6.05), 1.43 (1.01-2.02) and 2.39 (1.32-4.33), retinopathy: 4.41 (2.03-9.57), 1.77 (1.17-2.68) and 3.72 (1.85-7.47) and autonomic dysfunction: 3.25 (1.65-6.41), 1.64 (1.12-2.39) and 2.89 (1.54-5.42).

**Conclusions:**

24 h-CASP and 24 h-CPP was higher in patients *vs.* controls and increased with diabetic complications independently of covariates. Furthermore, 24 h-CASP was stronger associated to complications than 24 h-SBP.

The prognostic significance of 24 h-CASP and 24 h-CPP needs to be determined in follow-up studies.

**Trial registration:**

ClinicalTrials.gov ID NCT01171248.

## Background

Blood pressure (BP) control is of paramount importance in prevention of complications in type 1 diabetes [1-3]. Despite improved medical therapy over recent decades, complications still impact many diabetic patients diminishing quality of life and contributing to premature mortality. Moreover, monitoring and managing complications in patients with type 1 diabetes confers significant healthcare expenditure [4], and improved screening, monitoring and prevention strategies are warranted.

Routinely, seated BP measurements are recorded over the brachial artery in the physician’s office. However, increasing evidence points towards superior predictive value of out-of-office measurements such as 24 hour ambulatory BP (AMBP) [5] and home BP monitoring [6]. Recent studies indicate additional benefit from non-invasive assessment of central aortic systolic pressure (CASP) and central pulse pressure (CPP) as compared to routine measurement of brachial pressure [7-9].

Central and brachial pressures are mutually influenced by cardiac output, vascular resistance and conduit artery rigidity. Whilst mean arterial pressure (MAP) is relatively constant across large arteries, systolic pressure waves are amplified as they move from the aortic root to the brachial artery. This amplification results in a higher brachial systolic BP (SBP) relative to the corresponding CASP [10]. With aging [11] and/or diabetes [12] alterations in large artery composition associated with increased arterial stiffness, augmented blood flow and increased pressure wave reflections, impact more on CASP relative to SBP. In addition, antihypertensive treatment (AHT) may alter the relationship between CASP and SBP, resulting in different effects on CASP relative to SBP [13,14]. This might underpin different outcome benefits between classes of AHT drugs despite targeting to similar levels of SBP [15].

Measurement of ambulatory 24 h-CASP and 24 h-CPP are now feasible, and a recent study showed a difference in central and brachial diurnal patterns, which could imply that central BP possess additional prognostic value [16]. We investigate the associations between 24 h-CASP and 24 h-CPP, and impaired kidney function, history of cardiovascular disease (CVD), left ventricular hypertrophy (LVH), retinopathy, and autonomic dysfunction in patients with type 1 diabetes.

## Methods

### Study population

Between September 2009 and June 2011, Caucasian patients with TIDM, were recruited to enter a cross-sectional study at Steno Diabetes Center, Copenhagen, Denmark. Recruitment was in sequential order according to date of birth from a list of all patients attending the outpatient clinic. For power calculations we used an α-value of 5%, a β-value of 20%, and an estimated variance of 9.5 mmHg. For the comparison between controls and patients we estimated a 24 h-CASP-difference of 4 mmHg between groups, which implied that we needed a minimum of 45 participants in each group. For the comparisons between patient groups (3 albuminuria groups), we estimated a 24 h-CASP-difference of 2 mmHg, indicating a need for 177 patients in each group. Thus, we intended to include at least 45 controls and 576 patients. Of 1285 patients invited, 676 (52.6%) agreed to enter the study. Patients declining participation were younger but with similar gender distribution compared to participating patients (49 ± 16 years and 57% males *vs.* 54 ± 13 years and 56% males (*p *< 0.001 and 0.62). In addition, a control group of 51 non-diabetic subjects from Copenhagen, Denmark and 46 non-diabetic subjects from Leichester, UK were included.

**Figure 1:**
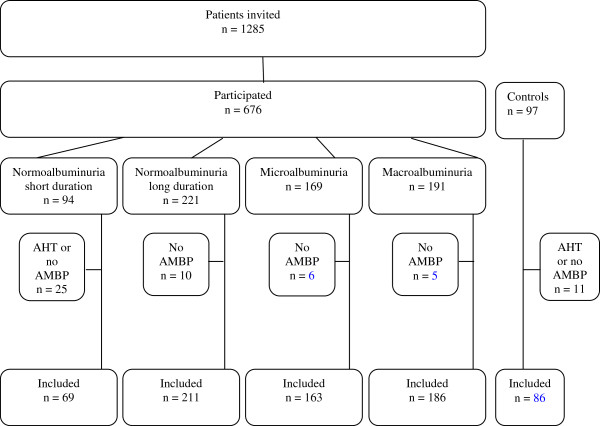
Flow chart of the cohort.

In total, 654 (96.7%) patients and 90 (92.9%) controls had adequate ambulatory AMBP available, with 46 ± 7 and 18 ± 3 (63.9% and 75.0% of total possible) recordings during day and night, respectively. Four controls and 25 patients with normoalbuminuria and short diabetes duration received AHT and were excluded in order to create two comparable groups not treated for hypertension. The remaining groups of patients had no restriction regarding AHT (Table 1). Hence, the analysed cohort included 715 subjects, comprising of 86 non-diabetic persons (C), 69 patients with short diabetes duration (< 10 years), normoalbuminuria (< 30 mg/24 h) and not receiving AHT (SN), 211 patients with longstanding diabetes (≥ 10 years) and normoalbuminuria (LN), 163 patients with microalbuminuria (30-299 mg/24 h) (Mi), and 186 patients with macroalbuminuria (> 300 mg/24 h) (Ma). Figure 1 summarise the inclusion and exclusion of the study participants. The 10 year cut-off for defining patients as having short *vs*. long duration of diabetes was chosen, as complications to diabetes may develop within 5–10 years [17]. The five groups were stratified in order to create groups for comparison of 1) healthy controls to patients without complications, 2) healthy controls to patients with sustained normal kidney function, and 3) patients with increasing degree of impaired kidney function.

**Table 1:** Baseline characteristics by group

	**All patients**	**Controls**	**Normoalbuminuria**		**Elevated albuminuria**		**Controls *vs. *short duration, normo-albuminuria**	**Long duration, normo- *vs. *micro- *vs. *macroalbuminuria**
			**Short duration**	**Long duration**	**Micro**	**Macro**	***p***	***p***
	**n = 629**	**n = 86**	**n = 69**	**n = 211**	**n = 163**	**n = 186**		
Female (%)	44	44	39	53	38	42	0.84	0.010
Age (years)	54 ± 13	49 ± 12	39 ± 13	57 ± 11	58 ± 13	55 ± 10	<0.001	0.031
Diabetes duration (years)	34 ± 15	n/a	6 ± 3	38 ± 11	35 ± 15	38 ± 11	N/A	0.056
HbA_1c_ (mmol/mol)	64 ± 13	35 ± 3	65 ± 13	62 ± 10	65 ± 13	68 ± 14	<0.001	<0.001
Total cholesterol (mmol/l)	4.7 ± 0.9	5.5 ± 0.9	4.7 ± 0.8	4.8 ± 0.7	4.6 ± 0.8	4.6 ± 1.0	<0.001	0.23
Body mass index (kg/m^2^)	25.5 ± 5.9	26.6 ± 5.2	24.2 ± 3.2	25.0 ± 3.8	25.8 ± 4.1	26.3 ± 9.1	0.001	0.045
eGFR (ml/min/1.73 m^2^)	83 ± 28	96 ± 16	107 ± 21	90 ± 20	85 ± 26	62 ± 29	<0.001	<0.001
^*^UAER (mg/24-h)	18 (2–8271)	14 (4–157)	10 (4–39)	7 (2–236)	33 (4–4512)	130 (4–8271)	0.36	<0.001
Smokers (%)	21	6	23	18	19	25	0.002	0.18
Antihypertensive treatment (%)	71	0	0	56	90	98	N/A	<0.001
Cardiovascular disease (%)	21	4	1	14	30	29	0.14	<0.001
Left ventricular hypertrophy (%)	4	7	7	4	4	4	0.91	0.82
Autonomic dysfunction (%)	60	7	7	53	64	83	0.48	<0.001
Retinopathy (%)	81	0	23	83	84	96	<0.001	<0.001

Data represents percentage (%), mean ± SD or median (range). P-values are for unadjusted comparisons (ANOVA) between groups.

^*^Some patients with previously persistent macroalbuminuria had values <300 mg/24 h at the time of investigation.

Patients with ESRD, defined as receiving dialysis or renal transplantation, or GFR/eGFR < 15 ml/min/1.73 m^2^ were not included.

The study conformed to the Declaration of Helsinki, was approved by the Danish National Committee on Biomedical Research Ethics (2009–056), and all participants gave written informed consent.

### Clinical and laboratory methods

Participant attended one single study visit, during which all clinical and laboratory measurements were performed. For office BP, 15 minutes supine rest was followed by recording and averaging of three separate left brachial measurements (A&D Medical, UA787).

AMBP were recorded non-invasively, using a validated system consisting of a radial arterial tonometer embedded in an articulating strap of a wrist-watch device (BPro™, HealthStats Singapore). The device was calibrated to brachial BP and variations in pulse waveform height over the subsequent measurement period used to calculate brachial AMBP [18]. Calibrated radial waveforms were then processed at the end of each measurement session by applying an N-point-moving average to derive CASP [19]. The device reported CASP, CPP, SBP, diastolic BP (DBP), MAP and heart rate (HR) every 15 minutes during 24 h. Previous validation studies have shown the BPro device to obtain BP measurements independently of arm positioning, with values comparable to those obtained with cuff-based devices [20,21]. Furthermore, we have shown that CASP measurements obtained by the BPro are comparable to those obtained by accepted gold standard devices for non-invasive measurements of CASP [22]. AMBP data was considered adequate if ≥ 14 and ≥ 7 recordings were obtained during day- and nighttime [23].

Following 15 minutes supine rest, office measurements of central BP were obtained with the SphygmoCor device (Atcor Medical, Sydney, Australia) by trained laboratory technicians according to guidelines.

Electrocardiographs (ECG) were recorded with Cardiosoft V6.51 (GE Healthcare, USA) and LVH defined according to Sokolow-Lyon, Cornell and/or Romhilt-Estes score [24] by the investigating M.D. Heart rate variability (HRV) was measured after 15 minutes supine rest, during paced deep breathing [25] to classify autonomic dysfunction. HRV was assessed by expiration/inspiration variation in heart rate. While resting in supine position, the patient was asked to breathe deeply at the rate of 6 breaths per minute for 1 minute, during which an electrocardiogram was recorded. Subsequently the means of the difference in highest and lowest heart rates (HR) during each breathing cycle were calculated. An abnormal value was < 11 beats per minute. Retinopathy status obtained from medical records, and assessed from retinal photographs, taken every 3–24 months, were classified as normal if no retinopathy was present, and abnormal if presence of simple or proliferative retinopathy or blindness.

All participants had blood samples and phenotypic characteristics collected. HbA_1c_ was measured by high-performance liquid chromatography (Variant; Biorad Laboratories, Germany), urinary albumin excretion rate (UAER) was measured in 24 h sterile urine collections by enzyme immunoassay, and plasma creatinine concentration by an enzymatic method (Hitachi 912, Roche Diagnostics, Germany).

Patients were stratified as normoalbuminuric if two out of three consecutive measurements contained normoalbuminuria with UAER < 30 mg/24 h, and micro- or macroalbuminuric if UAER was or previously recorded between 30–299 mg/24 h or above 300 mg/24 h, in two out of three consecutive measurements, respectively. Estimated GFR (eGFR) was calculated by the four variable Modification of Diet in Renal Disease (MDRD) formula. Based on standardized questionnaires, current daily use of ≥ 1 cigarettes/cigars/pipes classified smokers. Previous of CVD was myocardial infarction, stroke, or peripheral arterial disease based on standardized WHO questionnaires and patient records from Steno Diabetes Center.

### Statistical analysis

Normally distributed variables are given as mean ± SD. The non-normally distributed variable (UAER) is given as median (range) and log10 transformed before analysis. Comparisons between groups were performed by unpaired Student’s t-test or analysis of variance (ANOVA).

Univariate and mulitivariate linear regression compared hemodynamic variables with covariates. Analysis of covariance (ANCOVA) was applied for multivariable adjustment when comparing hemodynamic variables in different groups and in patients with or without complications. Adjustments were made for gender, age, 24 h-MAP, 24 h-HR, eGFR, HbA_1c_, smoking and antihypertensive treatment. In comparisons including controls HbA_1c_ and antihypertensive treatment was left out.

The multivariate logistic regression between hemodynamic variables and diabetic complications also included UAER and office CASP, CPP or SBP. Although for LVH the analyses were only adjusted for gender, age and 24 h-MAP.

A two-tailed *p*-value of < 0.05 was considered statistically significant. Statistical analyses were performed using SPSS for Windows, version 20.0 (SPSS, Chicago, IL).

## Results

### Baseline

Characteristics of the analysed cohort consisting of 629 patients and 86 controls are shown in Tables 1 and 2. Patients included and excluded from the analyses had similar age, diabetes duration, gender distribution, HbA_1c_, total cholesterol, eGFR, UAER, pulse wave velocity, HRV, frequency of previous CVD, retinopathy status and presence of LVH (*p* > 0.11). Table 3 represent the estimated marginal means adjusted for gender, age, 24 h-MAP, 24 h-HR, UAER, eGFR, HbA_1c_, smoking and antihypertensive treatment.

**Table 2:** Hemodynamic variables by group

	**Controls**	**Normoalbuminuria**		**Elevated albuminuria**		**Controls *vs. *short duration, normo-albuminuria**	**Long duration, normo- *vs. *micro- *vs. *macroalbuminuria**
		**Short duration**	**Long duration**	**Micro**	**Macro**	***p***	***p***
	**n = 86**	**n = 69**	**n = 211**	**n = 163**	**n = 186**		
24 h-CASP (mmHg)	114 ± 17	115 ± 13	121 ± 14	119 ± 16	121 ± 13	0.60	0.19
Daytime CASP (mmHg)	118 ± 18	119 ± 14	124 ± 14	122 ± 16	124 ± 13	0.59	0.26
Nighttime CASP (mmHg)	107 ± 16	108 ± 14	114 ± 13	112 ± 16	116 ± 14	0.68	0.029
Office CASP (mmHg)	117 ± 20	112 ± 14	118 ± 18	120 ± 18	120 ± 20	0.057	0.079
24 h-CPP (mmHg)	38 ± 8	38 ± 7	44 ± 10	46 ± 11	46 ± 11	0.96	0.14
Daytime CPP (mmHg)	39 ± 8	39 ± 8	45 ± 10	46 ± 11	46 ± 11	0.59	0.13
Nighttime CPP (mmHg)	36 ± 8	36 ± 7	42 ± 10	44 ± 11	45 ± 11	0.68	0.059
24 h brachial SBP (mmHg)	122 ± 19	124 ± 15	129 ± 15	127 ± 17	131 ± 15	0.43	0.18
Daytime brachial SBP (mmHg)	127 ± 19	129 ± 15	134 ± 15	132 ± 17	134 ± 15	0.37	0.24
Nighttime brachial SBP (mmHg)	113 ± 19	115 ± 15	121 ± 14	119 ± 17	124 ± 15	0.52	0.043
Mean arterial pressure (mmHg)	91 ± 14	93 ± 11	94 ± 10	91 ± 10	94 ± 11	0.43	0.011
24 h heart rate (beats per minute)	66 ± 7	67 ± 7	70 ± 8	69 ± 9	74 ± 10	0.31	<0.001

Data are mean ± SD.

*p*-values are for unadjusted comparisons (ANOVA) for comparison of controls to patients with short duration of normoalbuminuria or between patients with longstanding normoalbuminuria, microalbuminuria and macroalbuminuria.

CASP: central aortic systolic pressure, CPP: central pulse pressure, SBP: systolic blood pressure.

**Table 3:** Blood pressure variables given as adjusted estimated means for type 1 diabetes patients

	**Controls**	**Normoalbuminuria**		**Elevated albuminuria**		**Controls *vs. *short duration, normo-albuminuria**	**Long duration, normo-*vs. *micro- *vs. *macroalbuminuria**
		**Short duration**	**Long duration**	**Micro**	**Macro**	***p***	***p***
	**n = 86**	**n = 69**	**n = 211**	**n = 163**	**n = 186**		
24 h-CASP (mmHg)	117 (116–119)	118 (117–119)	119 (118–120)	121 (120–121)	122 (121–122)	0.39	<0.001
Daytime CASP (mmHg)	122 (120–123)	122 (121–123)	121 (121–122)	123 (123–124)	125 (124–126)	0.60	<0.001
Nighttime CASP (mmHg)	109 (107–111)	110 (109–112)	112 (111–114)	114 (113–115)	116 (115–117)	0.30	<0.001
Office CASP (mmHg)	110 (107–112)	111 (109–113)	119 (117–121)	120 (118–122)	118 (115–120)	0.44	0.41
24 h-CPP (mmHg)	38 (36–40)	39 (37–41)	43 (42–44)	46 (44–47)	47 (46–49)	0.59	<0.001
Daytime CPP (mmHg)	39 (37–41)	40 (38–41)	43 (42–45)	46 (45–48)	48 (46–49)	0.74	<0.001
Nighttime CPP (mmHg)	36 (34–38)	37 (35–39)	41 (40–43)	44 (43–45)	46 (44–47)	0.54	<0.001
24 h brachial SBP (mmHg)	126 (125–128)	126 (125–128)	128 (126–129)	130 (127–131)	131 (130–132)	0.98	<0.001
Daytime brachial SBP (mmHg)	132 (129–134)	132 (130–134)	132 (130–134)	134 (133–135)	135 (134–136)	0.90	0.001
Nighttime brachial SBP (mmHg)	116 (114–118)	117 (115–119)	119 (118–121)	122 (120–123)	123 (122–125)	0.56	0.001
24 h-MAP (mmHg)	92 (89–95)	92 (90–95)	95 (94–97)	92 (90–93)	92 (90–94)	0.80	0.004
24 h heart rate (beats per minute)	65 (63–67)	67 (66–69)	72 (70–73)	70 (68–71)	73 (71–74)	0.08	0.005

Estimated marginal means with 95% confidence intervals. P-values are for adjusted comparisons (ANCOVA) of controls to patients with short duration of normoalbuminuria or for comparison between patients with longstanding normoalbuminuria, microalbuminuria and macroalbuminuria. Adjustments were made for gender, age, 24 h-MAP (except for 24 h-MAP), 24 h HR, eGFR and smoking. Adjustments also included HbA_1c_ and antihypertensive treatment when comparing patients with normoalbuminuria and long diabetes duration, microalbuminuria and macroalbuminuria.

CASP: central aortic systolic pressure, CPP: central pulse pressure, SBP: systolic blood pressure, MAP: mean arterial pressure, microalbuminuria: 30-299 mg/24 h, macroalbuminuria > 300 mg/24 h.

Correlations between covariates and 24 h-CASP, 24 h-CPP and 24 h-SBP in all participants are shown in Table 4. In short, all three blood pressures correlated with age, diabetes duration, 24 h-MAP, 24 h-HR, UAER and eGFR. Furthermore, 24 h-CPP correlated with total cholesterol, 24 h-SBP was higher in men, and all three blood pressures were higher in patients receiving AHT. None of the three blood pressures correlated with HbA_1c_ or body mass index.

**Table 4:** Correlations between 24 h-CASP, 24 h brachial SBP or 24 h-CPP and covariates in all participants (n = 715)

	**24 h brachial SBP**	**24 h-CASP**	**24 h-CPP**
Office CASP	r = 0.64, *p* < 0.001	r = 0.67, *p* < 0.001	r = 0.56, *p* < 0.001
24 h-MAP	N/A*	r = 0.90, *p* < 0.001	r = 0.32, *p* < 0.001
24 h-heart rate	r = 0.12, *p* = 0.002	0.17	r = 0.13, *p* < 0.001
logUAER	r = 0.18, *p* < 0.001	r = 0.17, *p* < 0.001	r = 0.18, *p* < 0.001
Diabetes duration	r = 0.13, *p* = 0.01	r = 0.12, *p* = 0.001	r = 0.40, *p* < 0.001
Age	r = 0.11, *p* = 0.03	r = 0.12, *p* = 0.001	r = 0.40, *p* < 0.001
eGFR	r = 0.12, *p* = 0.02	r = 0.12, *p* = 0.02	r = 0.23, *p* < 0.001
HbA_1c_	0.71	0.78	0.94
Total cholesterol	0.11	0.10	r = 0.08, *p* = 0.039
Body mass index	0.30	0.38	0.71
Gender	Male > female, *p* = 0.02	0.21	0.19
Antihypertensive treatment	Highest in treated patients, *p* < 0.001	Highest in treated patients, *p* < 0.001	Highest in treated patients, *p* < 0.001
Smoking	0.43	0.65	0.46

r-values are Pearson’s coefficients.

MAP: mean arterial pressure, UAER: urinary albumin excretion rate, eGFR: estimated glomerular filtration rate.

* Since SBP is included in MAP.

In adjusted analyses only age, diabetes duration, 24 h-MAP, 24 h-HR and UAER correlated with 24 h-CASP, 24 h-CPP and 24 h-SBP (*p* ≤ 0.001).

**Figure 2:**
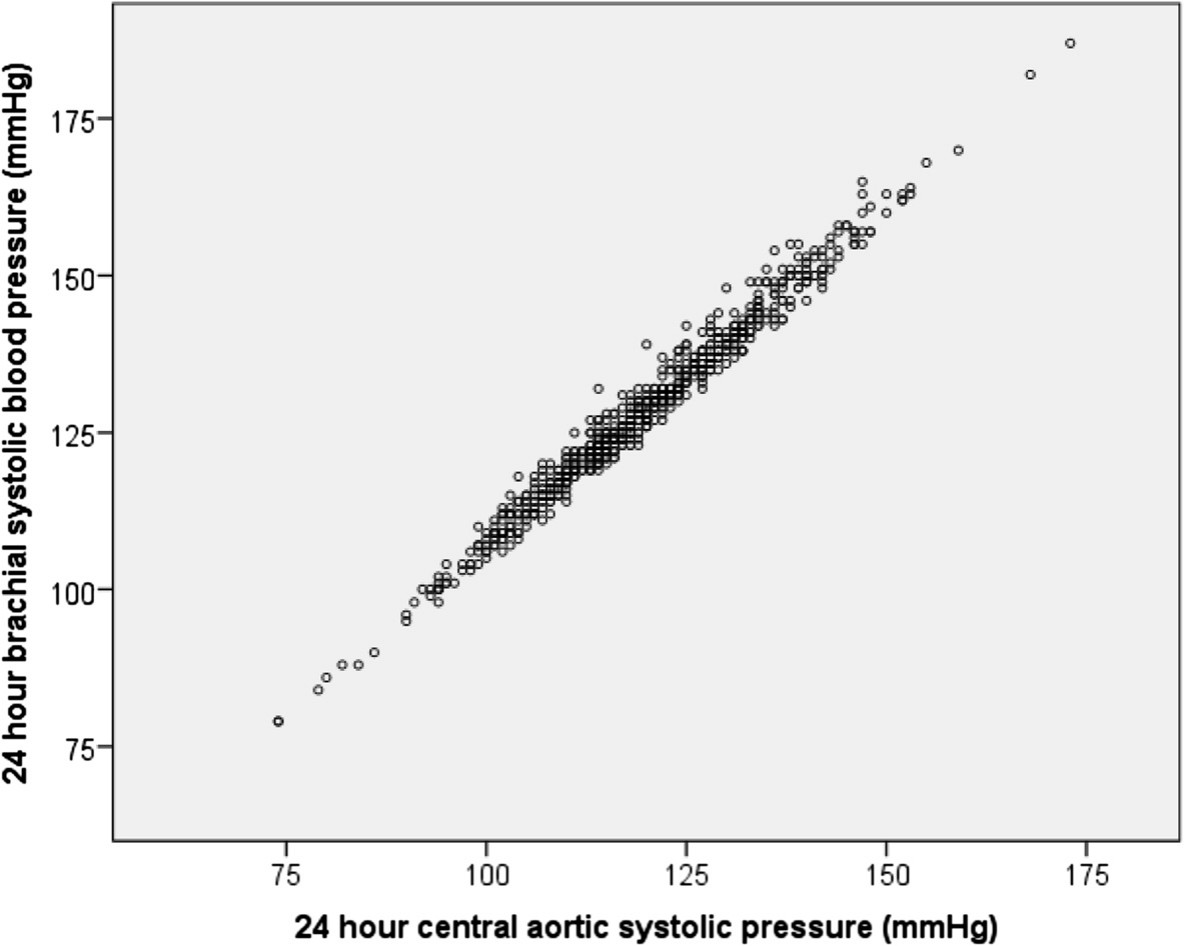
**Scatter plot for 24 h-CASP and 24 h-SBP.** R = 0.99, *p* < 0.001.

Correlations between 24 h-CASP and 24-SBP were high (r = 0.99, *p* < 0.001 for 24 h, daytime and nighttime BPs). For comparison, the correlation between office CASP measured by SphygmoCor and supine brachial SBP was also high (r = 0.96, *p* < 0.001), indicative of an overall close relation between CASP and brachial SBP. However, despite the close correlation, there was a scatter between central and brachial measurements as seen in Figure 2. Moreover, correlations between 24 h-CPP and 24 h-SBP was much lower (r = 0.72, *p* < 0.001 for 24 h, daytime and nighttime BPs). As was the correlation between 24 h-CASP and office CASP (r = 0.67, *p* < 0.001).

### 24 h-CASP and 24 h-CPP comparison between groups

C and SN had similar 24 h-CASP and 24 h-CPP (unadjusted *p* = 0.60 and 0.96; adjusted *p* = 0.39 and 0.59) (Tables 2 and 3). Including further adjustment for body mass index and total cholesterol did not alter these results (*p* ≥ 0.61). Controls compared to all normoalbuminuric patients (SN and LN) had lower 24 h-CASP and 24 h-CPP (38 ± 8 and 114 ± 17 *vs.* 43 ± 10 and 119 ± 14 mmHg, respectively) (*p* < 0.001 and 0.004; adjusted *p* < 0.001 for both). Additional adjustment for body mass index and total cholesterol did not alter these results (*p* = 0.016 and 0.018).

Between the groups with LN, Mi, and Ma, 24 h-CASP was lowest in Mi albeit not statistically significant (*p =* 0.19), while 24 h-CPP was almost similar between groups (*p* = 0.14) (Table 2). Following adjustment, both 24 h-CASP and 24 h-CPP was associated with increased albuminuria group (*p <* 0.001 for both) (Table 3). If investigating level of albuminuria as a continuous variable in all patients, both 24 h-CASP and 24 h-CPP were independently associated with UAER (*p* ≤ 0.001)

### 24 h-CASP, 24 h-CPP and 24 h-SBP in patients with *vs*. without history of CVD

The 24 h-CASP, 24 h-CPP and 24 h-SBP in patients with (n = 134 (21.3%)) *vs.* without a history of CVD was 121 ± 14, 49 ± 11 and 127 ± 16 *vs.* 119 ± 14, 43 ± 10 and 129 ± 16 mmHg (*p* = 0.43, < 0.001 and = 0.18). Following adjustment, 24 h-CASP, 24 h-CPP and 24-h SBP was higher in patients with a history of CVD (*p* < 0.001 for all). Per 1 standard deviation (SD) increase in 24 h-CASP, 24 h-CPP and 24-h SBP the odds ratio (OR) for previous CVD was 3.2 (1.7-6.1), 1.4 (1.0-2.0) and 2.4 (1.3-4.3) (*p* < 0.001, = 0.045 and = 0.004) (adjusted for gender, age, 24 h-MAP, 24 h-HR, UAER, eGFR, HbA_1c_, smoking, AHT, and either office CASP, office CPP or office SBP (Table 5). If total cholesterol and body mass index was further included in the adjusted model, *p*-values for a history of CVD were 0.001 for 24 h-CASP, 0.053 for 24 h-CPP and 0.05 for 24 h-SBP.

**Table 5:** Adjusted odds ratios between hemodynamic variables and diabetic complications

**Complications**	**Patients with disease/ without disease**	**24 h-CASP (1 SD = 14.2 mmHg)**	**24 h-CPP (1 SD = 10.7 mmHg)**	**24 h-SBP (1 SD = 15.6 mmHg)**
Cardiovascular disease^*^	n = 134/495	3.19 (1.68-6.05)^‡^	1.43 (1.01-2.02)^§^	2.39 (1.32-4.33)^§^
Left ventricular hypertrophy^†^	n = 27/602	2.51 (0.98-6.45)	1.50 (0.98-2.30)	2.14 (0.92-5.01)
Retinopathy^*^	n = 507/122	4.41 (2.03-9.57)^‡^	1.77 (1.17-2.68)^§^	3.72 (1.85-7.47)^‡^
Autonomic dysfunction^*^	n = 350/237	3.25 (1.65-6.41)^§^	1.64 (1.12-2.639)^§^	2.89 (1.54-5.42)^§^

Values are odds ratios and 95% confidence intervals. Odds ratios are per 1 standard deviation (SD) increase in blood pressure.

^*^Adjusted for gender, age, 24 h-MAP, 24 h-HR urinary albumin excretion rate, estimated glomerular filtration rate, HbA_1c_, smoking and antihypertensive treatment. 24 h-CASP is also adjusted for office CASP, 24 h-CPP for office CPP and 24 h-SBP for office SBP.

^†^Adjusted for gender, age and 24 h-MAP due to low frequency.

^‡^*p* < 0.001, ^§^*p* < 0.05

### 24 h-CASP, 24 h-CPP and 24 h-SBP in patients with *vs.* without LVH

The 24 h-CASP, 24 h-CPP and 24 h-SBP in patients with (n = 27 (4.3%)) *vs.* without LVH were 125 ± 11, 48 ± 11 and 132 ± 15 *vs.* 119 ± 14, 44 ± 10 and 128 ± 16 mmHg (*p* = 0.001, 0.019 and 0.13). Following adjustment for gender, age and 24 h-MAP the significance attenuated (*p* = 0.09, 0.10 and 0.06). Per 1 SD increase in 24 h-CASP, 24 h-CPP and 24 h-SBP, the OR for LVH was 2.5 (1.0-6.5), 1.5 (1.0-2.3) and 2.1 (0.9-5.0) (*p* = 0.057, 0.061, and 0.079) (Table 5).

### 24 h-CASP, 24 h-CPP and 24 h-SBP in patients with *vs.* without retinopathy

In patients with (n = 507 (80.6%)) *vs.* without retinopathy, 24 h-CASP, 24 h-CPP and 24 h-SBP was 121 ± 14, 46 ± 11 and 130 ± 15 *vs*. 115 ± 14, 39 ± 8 and 124 ± 15 mmHg (*p* < 0.001 for all). Following adjustment all three BP’s were higher in patients with retinopathy (*p* = 0.001 for all). Per 1 SD increase in 24 h-CASP, 24 h-CPP and 24 h-SBP, the OR for presence of retinopathy was 4.4 (2.0-9.6), 1.8 (1.2-2.7) and 3.7 (1.9-7.5) (*p* < 0.001 for all) (Table 5). If including total cholesterol and body mass index in the adjusted model, *p*-values for retinopathy were < 0.001 for 24 h-CASP, 0.004 for 24 h-CPP and < 0.001 for 24 h-SBP.

### 24 h-CASP, 24 h-CPP and 24 h-SBP in patients with *vs.* without autonomic dysfunction

Autonomic dysfunction was present in 350 (59.4%) of 587 assessed patients. Patients with *vs.* without autonomic dysfunction had 24 h-CASP, 24 h-CPP and 24 h-SBP of 121 ± 14, 47 ± 11 and 130 ± 16 *vs.* 117 ± 12, 40 ± 8 and 126 ± 13 mmHg (*p* < 0.001 for all). Following adjustment, 24 h-CASP, 24 h-CPP and 24 h-SBP remained significantly higher in patients with autonomic dysfunction (*p* = 0.001 for all). Per 1 SD increase in 24 h-CASP, 24 h-CPP and 24 h-SBP the OR for presence of autonomic dysfunction was 3.3 (1.7-6.4), 1.6 (1.1-2.4) and 2.9 (1.5-5.4) (*p =* 0.001 for all) (Table 5). If we further included total cholesterol and body mass index in the adjusted model, *p*-values for CVD were 0.001, 0.006 and 0.002 for 24 h-CASP, 24 h-CPP and 24 h-SBP, respectively.

## Discussion

This is the first study to measure 24 h-CASP and 24 h-CPP in patients with type 1 diabetes. The adjusted values of 24 h-CASP and 24 h-CPP was lower in controls compared to normoalbuminuric patients, and associated with increasing degree of albuminuria. Furthermore, 24 h-CASP and 24 h-CPP was higher in patients with a history of CVD, retinopathy and autonomic dysfunction, but similar in patients with and without LVH on ECG. Importantly, the positive associations were present despite rigorous adjustment for baseline characteristics including brachial 24 h-MAP. If we further included office CASP or office CPP in the adjustments, both 24 h-CASP and 24 h-CPP remained significantly (*p* ≤ 0.045) associated with all complications except albuminuria (data not shown), suggesting additional risk information of 24 hour measurements as compared to office measurement. Furthermore, 24 h-CASP was closer associated to diabetic complications as compared to 24 h-SBP.

As expected, normoalbuminuric type 1 diabetes patients had higher 24 h-CASP and 24 h-CPP than controls. Patients with type 1 diabetes are known to develop accelerated arterial stiffness [26] caused by multifactorial mechanisms including increased production of advance glycation end products [27] and increased oxidative stress [28]. Furthermore, insulin resistance impairs the ability of insulin to decrease pressure augmentation and CASP [29]. However, controls and normoalbuminuric patients with short diabetes duration had similar 24 h-CASP and 24 h-CPP, consistent with the likelihood that arterial damage caused by diabetes is a later complication, not appearing until > 10 years of diabetes duration.

Increasing degree of albuminuria was independently associated with higher 24 h-CASP and 24 h-CPP. We expected this, as albuminuria has been shown to be associated with arterial stiffness [30]. However, our study is the first to measure 24 h-CASP and 24 h-CPP in patients with type 1 diabetes and to demonstrate a relationship between albuminuria and both 24 h-CASP and 24 h-CPP, independent of MAP, kidney function and conventional risk factors.

Presence of CVD was independently associated with higher 24 h-CASP and 24 h-CPP. CASP reflects the afterload on the heart, and as such, it may be a superior indicator of cardiovascular health, as indicated by a closer association with the risk of CVD and to constitute a better treatment target than brachial BP. Increasing CPP results from a combination of higher CASP and lower diastolic central BP. As cardiac perfusion occurs in diastole it is conceivable, that a higher CPP will compromise perfusion, which could indicate that CPP may also be a strong CVD risk marker.

An association between retinopathy and central BP has never before been investigated, although others have shown elevated brachial BP (> 130/90 mmHg) in type 2 diabetes to influence development of retinopathy [31] and high brachial BP (> 150/90 mmHg) in type 1 diabetes to enhance progression of retinopathy [32]. We now show an independent association with central BP and retinopathy in type 1 diabetes patients.

Longer diabetes duration with presence of autonomic dysfunction does affect BP regulation. This is partly due to autonomic dysfunction being associated with altered vascular adrenoceptor sensitivity and catecholamine release [33,34], altered renin release [35] and the concurrent development of arterial stiffness [36] and other diabetic complications [25]. Thus, by demonstrating an association between autonomic dysfunction and 24 h central BP our data concurs with and extends previous findings.

The close correlation between 24 h-CASP and 24 h-SBP is understandable, as both were calculated from the same radial pulse wave. Given this close correlation, 24 h-CASP may not be superior to 24 h-SBP. However, we show that measurements of 24 h-CASP are feasible, and that values are strongly associated with diabetic complications, independently of brachial 24 h-MAP. Furthermore, we also exposed 24 h-CPP to be associated with diabetic complications, and the correlation between 24 h-CPP and 24 h-SBP was much weaker. Thus, despite the correlations between central and brachial BPs, the former may offer additional risk predictive value. This is in accordance with other studies, which have documented central BP to be closer associated with end organ damage [37] and superior to brachial BP in predicting outcome [8,9], although, this was not confirmed in a recent meta-analysis [8]. Furthermore, central BP is associated with left ventricular mass in type 2 diabetes [38] and with left ventricular function in patients with abnormal diastolic function [39], commonly present in diabetes [40]. This is in accordance with our finding of higher 24 h-CASP in patients with LVH, although the significance attenuated on adjustment, which may however be due to lack of power.

In a future perspective, the 24 h-CASP and 24 h-CPP may offer additional predictive value to 24 h-SBP in patients with type 1 diabetes. However, whether these methods of evaluating central stiffness will add prognostic information or, if there is any prognosis superiority between them, can only be determined in prospective studies. If subtle differences in 24 h-CASP and 24 h-SBP impact on outcome, targeting control of CASP rather than SBP may be more efficient in prevention of complications. The CAFE study, a sub-study of the ASCOT trial, investigated the effect of reducing CASP, and showed calcium blockers and beta blockers to exhibit different effect on CASP despite similar effect on SBP [13]. Another study showed reductions in B-type natriuretic peptide (a peptide secreted by the ventricles in response to strain and a marker of heart failure) in patients treated with nicorandil, likely secondary to reduced CASP [41], suggesting risk reduction associated with decreased CASP. Finally, a recent study demonstrated a reduction in left ventricular mass following treatment with perindopril and indapamide [42], which could be attributable to reduced CASP [39].

We suggest that CASP and CPP may supplement the predictive value of brachial BP in patients with type 1 diabetes. Moreover, central BP could be a future target for BP monitoring and control. Although, follow-up of this and other future prospective studies are required to verify if modifying CASP and/or CPP may supplement or even exceed the predictive value of modifying brachial BP on diabetic complications.

### Strengths and limitations

The 24 h-CASP and 24 h-CPP was measured by a wrist bound tonometric device. This device has previously been validated with other non-invasive and invasive measurements [19,21,22]. More recently, Komori et al. showed that arm positioning did not affect BP measurements by the BPro device [20]. Adjustment were made for 24 h-MAP rather than 24 h-SBP, -DBP or pulse pressure (PP) -PP, as MAP represents steady components of the BP, while SBP and PP contain pulsatile components and represent surrogates for arterial stiffness. Furthermore, 24 h-SBP, 24 h-CASP and 24 h-CPP were derived from the same pulse wave. Participants in the control group were compared to patients with short diabetes duration and normoalbuminuria. They were not fully matched on several covariates, including age. This could account for the lack of differences in the blood pressures between these two groups. We did however perform aggressive adjustments to compensate for this. The groups of patients with *vs.* without LVH were different in size, which limits the statistical power of these analyses. Furthermore, the diagnosis of LVH was solely based on ECG’s, which has a lower specificity and sensitivity than cardiac imaging.

We did adjust for antihypertensive treatment in general. However, it was not possible to compare patients receiving different single agent regiments, as most patients received several different agents.

Major strengths are the remarkably high sample size, and the study being from a single center, why the cohort was likely rather homogenous and receiving similar treatment. The majority of patients had been followed at the Steno Diabetes Center for more than a decade, rendering the information on diabetic complications very reliable.

## Conclusions

This is the first study to measure 24 h-CASP and 24 h-CPP in patients with type 1 diabetes. The 24 h-CASP and 24 h-CPP was increased in patients with type 1 diabetes compared to controls and increased with albuminuria, CVD, retinopathy and autonomic dysfunction independently of covariates. Furthermore, 24 h-CASP appeared to be stronger associated with complications as compared to 24 h-SBP. As measurement of 24 h ambulatory central BP is now feasible, further studies are required to determine its prognostic significance in patients with diabetes.

## Abbreviations

AHT: Antihypertensive treatment; AMBP: 24-hour ambulatory blood pressure; BPro: BPro HealthStats; CVD: Cardiovascular disease; CASP: Central aortic systolic pressure; CPP: Central pulse pressure; DBP: Diastolic blood pressure; ECG: Electrocardiogram; eGFR: Estimated glomerular filtration rate; HR: Heart rate; MAP: Mean arterial pressure; PP: Pulse pressure; SBP: Systolic blood pressure; UAER: Urinary albumin excretion rate.

## Competing interests

Williams is a National Institutes for Health Research (NIHR) Senior Investigator, supported by the University College London NIHR Biomedical Research Centre, London UK. Williams and Lacy have worked in scientific collaboration with HealthStats, Singapore developing methods for non-invasive derivation of central pressures from radial artery wave forms. Theilade, Lajer, Hansen, Joergensen, Persson, Andrésdottir, Reinhard, Nielsen and Rossing have received no grants/support and have nothing to disclose and no conflicts of interest.

## Authors’ contributions

ST researched and analysed the data, contributed to the discussion, wrote and edited the manuscript. ST is the guarantor for the manuscript. ML researched the data, contributed to the discussion and reviewed the manuscript. TWH analysed the data, contributed to the discussion, edited and reviewed the manuscript. CJ, FP and HR researched the data and reviewed the manuscript. GA and SEN researched the data. PL and BW contributed to the data analyses, and the discussion and edited the manuscript. PR researched and analysed the data, contributed to the discussion and reviewed the manuscript. All authors read and approved the final manuscript.
